# Neutrophil extracellular traps and severe mycoplasma pneumonia: A clinical observational study

**DOI:** 10.1097/MD.0000000000048645

**Published:** 2026-05-08

**Authors:** Zhonghua Hu, Lina Qi, Lifen Yuan, Baoxi Zhao, Junsheng Jiang

**Affiliations:** a Department of Pediatrics, The Second Affiliated Hospital of Zhejiang University, Linping Campus, Hangzhou, China.

**Keywords:** C-reactive protein, children, lactate dehydrogenase, Mycoplasma pneumoniae pneumonia, neutrophil extracellular traps, severity prediction

## Abstract

**Background:**

This full-scale confirmatory study aimed to measure serum neutrophil extracellular traps (NETs) levels in children with severe Mycoplasma pneumoniae pneumonia (SMPP) and non-severe MPP, and to compare the predictive performance of serum NETs, C-reactive protein (CRP), and lactate dehydrogenase (LDH) for SMPP.

**Methods:**

A total of 200 children hospitalized with MPP between January 2023 and August 2024 were consecutively enrolled and divided into SMPP (n = 100) and non-severe MPP (n = 100) groups. Baseline demographic and laboratory indices were compared between groups. Receiver operating characteristic (ROC) curves were used to assess predictive value, and univariate and multivariate logistic regression were performed to identify independent predictors of SMPP.

**Results:**

Age and sex distributions were comparable between groups (all *P* > .05). Serum white blood cell count, neutrophil ratio, CRP, D-dimer, LDH, and NETs were significantly higher in the SMPP group (all *P* < .001). ROC analysis showed serum NETs had the highest area under the curve (AUC = 0.83, 95% confidence interval [CI]: 0.762–0.896), with a cutoff value of 19.2, sensitivity 0.818, and specificity 0.835. Multivariate logistic regression confirmed serum NETs (odds ratio [OR] = 4.32, 95% CI: 3.45–7.25, *P* = .023), CRP (OR = 3.62, 95% CI: 1.85–9.25, *P* = .010), and LDH (OR = 1.63, 95% CI: 1.18–2.26, *P* = .001) as independent predictors of SMPP.

**Conclusion:**

Serum NETs are strongly elevated in children with SMPP and show better predictive performance than conventional CRP and LDH. Combined measurement of NETs, CRP, and LDH provides a reliable tool for the early identification of pediatric SMPP. This study extends and validates our preliminary exploratory findings with a larger sample and complete statistical analysis.

## 1. Introduction

Mycoplasma pneumoniae pneumonia (MPP) remains one of the most common community-acquired respiratory infections affecting children worldwide, imposing a substantial burden on pediatric healthcare systems.^[1,2]^ Notably, in the post-COVID-19 pandemic era over the past 2 years, the incidence of MPP among children has experienced a marked rebound, accompanied by a significant increase in the proportion of drug-resistant Mycoplasma strains in outpatient settings.^[3]^ This resurgence has brought great challenges to clinical practice, as clinicians are increasingly required to rapidly identify the disease and accurately evaluate its severity to guide timely and effective treatment. With the continuous emergence and spread of macrolide-resistant Mycoplasma pneumoniae, the incidence of severe Mycoplasma pneumoniae pneumonia (SMPP) has been on a steady annual rise.^[4]^ SMPP is not only associated with more severe pulmonary lesions but also carries a higher risk of various extrapulmonary complications, which can progress to multiple organ dysfunction syndrome and seriously endanger children’s life and long-term health.^[5]^ Thus, exploring reliable diagnostic indicators and scientific severity assessment methods is essential to avoid misdiagnosis, reduce irrational use of antibiotics, and mitigate the development of drug resistance, which is of great clinical significance for improving the prognosis of children with MPP.

Neutrophils are the first line of defense in the body’s innate immune response against microbial infections, and their functional abnormalities are closely related to the progression and severity of infectious diseases. In recent years, with in-depth research on neutrophil biology, neutrophil extracellular traps (NETs) have become a research hotspot in the field of immune regulation. NETs are web-like structures released by activated neutrophils, composed of decondensed chromatin and granular proteins, which can effectively trap and kill invading pathogens. However, accumulating evidence has shown that NETs play a complex dual role in respiratory infections: while appropriate NET formation contributes to pathogen clearance and disease control, excessive or dysregulated NET release (NETosis) can induce oxidative stress and inflammatory responses, leading to damage to surrounding tissues and organs. In the pathological process of MPP, Mycoplasma pneumoniae, as a wall-less pathogen, can escape the phagocytic clearance of neutrophils through multiple mechanisms, thereby triggering abnormal activation of neutrophils and alternative antimicrobial responses such as NETosis. Previous studies have confirmed that Mycoplasma pneumoniae can activate neutrophils through the Toll-like receptor 2 (TLR2) and nucleotide-binding oligomerization domain-containing protein 2 (NOD2) signaling pathways, which are key inducers of NET formation.^[6,7]^ This provides a clear theoretical basis for the involvement of NETs in the pathogenesis of MPP and its progression to severity.

Earlier studies have demonstrated that the formation of NETs is an important antibacterial mechanism of neutrophils,^[8]^ but recent studies have further found that abnormal NET activation is closely related to the occurrence and development of various inflammatory and infectious diseases, as well as autoimmune diseases and tumor metastasis. However, the specific role of NETs in the progression of MPP to severity, and whether NET levels can serve as potential indicators for evaluating MPP severity, remain unclear. To address this research gap, this study detected the expression levels of NETs in the serum of children with non-severe MPP and SMPP, as well as in the bronchoalveolar lavage fluid (BALF) from affected and unaffected lung segments of children with SMPP. By analyzing the differences in NET levels among different groups, we aimed to clarify the correlation between NETs and the severity of childhood MPP, and to provide new theoretical basis and clinical reference for the early identification, severity assessment, and targeted treatment of SMPP.

## 2. Materials and methods

### 2.1. Research subjects

From January 2023 to August 2024, a prospective observational investigation was implemented at The Second Affiliated Hospital of Zhejiang University, Linping Campus. All participating children were divided into 2 cohorts: a non-severe mycoplasma pneumonia group (MPP group, n = 100) and a severe mycoplasma pneumonia group (SMPP group, n = 100). No bronchoalveolar lavage fluid (BALF) specimens were collected from participants in the MPP group. On the other hand, 10 BALF samples were gathered from the SMPP group; imaging tests indicated that these children had unilateral lung affection, and BALF samples were therefore collected from both the diseased and normal lung regions. The study design was approved by the Clinical Research Ethics Committee ofThe Second Affiliated Hospital of Zhejiang University (Approval No. 2024048), which is in line with the ethical principles specified in the Declaration of Helsinki. Prior to the children’s participation in the study, written informed consent was obtained from their respective legal guardians.

### 2.2. Inclusion and exclusion criteria

Inclusion criteria: Children aged 0 to 14 years old; diagnosis consistent with the criteria for MPP and SMPP (as specified in the “Expert Consensus on the Diagnosis and Treatment of Mycoplasma Pneumonia in Children (2023 Edition)”); the course of the disease does not exceed 14 days; and blood culture results are negative, and there is no clear indication of viral infection.

Exclusion criteria: Children with a past medical history of bronchial asthma or chronic obstructive pulmonary disease; children with severe underlying conditions, such as hematological diseases, severe congenital heart disease, severe hepatic and renal insufficiency, congenital bronchopulmonary dysplasia, genetic metabolic disorders, and immunodeficiency diseases; children who have undergone long-term therapy with glucocorticoids, immunomodulatory agents, or immunosuppressive drugs; and concurrent infection with other pathogenic organisms.

### 2.3. Methods

Sample size calculation was conducted with a 2-sided α of 0.05 and a statistical power (1-β) of 90%. The final sample sizes (100 subjects in the MPP group and 100 subjects in the SMPP group) not only satisfied but also surpassed the calculated statistical demands, while also accommodating the clinical accessibility of participants during the study period.

Demographic information, including gender and age, was recorded for each enrolled child. Following hospital admission, 2 mL of fasting venous blood was drawn from each participant. Bronchoalveolar lavage was performed in accordance with the principle of prioritizing the healthy lung followed by the affected lung, with 5 mL of BALF collected from each lung. The collected samples were subjected to centrifugation, and the supernatant was isolated and preserved at −80°Cfor future testing.

Routine blood parameters and C-reactive protein (CRP) were detected using an automated hematology analyzer (model: XN-9000, Sysmex, Japan), with white blood cell count, neutrophil proportion, and CRP level recorded. All detections were performed in duplicate, and the average value was used for statistical analysis.

D-dimer concentration was measured via automatic latex agglutination immunoturbidimetric technique (kit: D-dimer Assay Kit, Nanjing Jiancheng Bioengineering Institute, China) in duplicate. LDH levels and NETs content in serum and BALF were detected by sandwich ELISA (high specificity/sensitivity) in triplicate (RSD > 10% required retesting). LDH (cat. E-EL-H0182c) and NETs (cat. E-EL-H7096) kits were from Elabscience (Wuhan, China), stored at 2–8°Cprotected from light. Brief ELISA procedure: add standards/samples to pre-coated wells (37°C, 90 minutes), wash, add biotin-labeled antibody (37°C, 60 minutes), wash, add streptavidin-HRP (37°C, 30 minutes), wash, add TMB substrate (37°C, dark, 15 minutes), terminate with stop solution, and measure OD value at 450 nm (microplate reader: Multiskan FC, Thermo Fisher Scientific, USA).

### 2.4. Data analyses and statistics

Statistical analyses were conducted utilizing SPSS 26.0 and GraphPad Prism 8.0 software packages. Continuous data that followed a normal distribution were presented as mean ± standard deviation, and the independent samples t-test was utilized for comparisons between the 2 groups. For comparisons of indicators between serum and BALF within the same group, the paired t-test was applied. Continuous data that did not conform to a normal distribution were expressed as median (interquartile range) [M (P25, P75)], and inter-group comparisons were performed using the Wilcoxon rank-sum test. Categorical data were presented as count (percentage) (n [%]), and the χ^2^ test was used to compare differences between groups. Pearson correlation analysis was employed to explore the correlation between BALF NETs and serum NETs in the SMPP group. Indicators that showed statistical significance in univariate analysis were selected for further multivariate logistic regression analysis. *P* < .05 was regarded as statistically significant.

## 3. Result

### 3.1. Baseline characteristics of the study population

**Figure 1.**
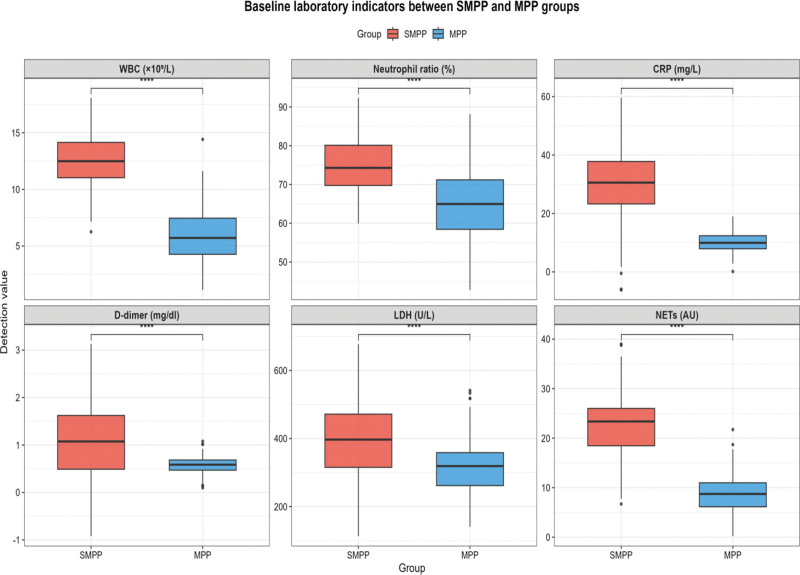
Baseline laboratory indicators between SMPP and MPP. MPP = Mycoplasma pneumoniae pneumonia, SMPP = severe Mycoplasma pneumoniae pneumonia.

A total of 200 children with MPP were enrolled in this study, including 100 cases in the SMPP group and 100 cases in the non-severe MPP group. As shown in Table 1, there were no statistically significant differences in gender distribution and age between the 2 groups, indicating that the baseline demographic data were balanced and comparable. However, the SMPP group exhibited significantly higher levels of serum laboratory indicators compared with the MPP group (*P* < .001): the WBC was 12.33 ± 2.63 × 10^9^/L in the SMPP group versus 6.28 ± 2.51 × 10^9^/L in the MPP group; the neutrophil ratio was 74 (64,80)% in the SMPP group versus 65 (54,72)% in the MPP group; the median C-reactive protein (CRP) level was 28.42 (16.58,42.53) mg/L in the SMPP group versus 10.25 (7.26,14.55) mg/L in the MPP group; the median D-dimer level was 1.16 mg/dl in the SMPP group versus 0.54 mg/dl in the MPP group; the LDH level was 385.7 ± 108.6 U/L in the SMPP group versus 321.2 ± 85.4 U/L in the MPP group; and the serum NETs level was significantly elevated in the SMPP group compared with the MPP group. The comparative distribution of these baseline laboratory indicators between the 2 groups was visually presented in Figure 1, which intuitively reflected the higher levels of WBC, neutrophil ratio, CRP, D-dimer, LDH, and NETs in the SMPP group.

**Table 1:** Baseline characteristics of the studied population.

	SMPP (n = 100)	MPP (n = 100)	χ ^2^/t/Z	*P*
Sex (male,%)	50 (50)	49 (49)	0.24	.650
Age, years	7.22 ± 2.55	7.93 ± 3.67	−0.57	.452
WBC (*10^9^/L)	12.33 ± 2.63	6.28 ± 2.51	3.52	.001
Neutrophil ratio (%)	74 (64,80)	65 (54,72)	4.26	.001
CRP (mg/L)	28.42 (16.58,42.53)	10.25 (7.26,14.55)	6.28	.001
D--dimer (mg/dl)	1.16 (0.67,2.75)	0.54 (0.34,0.74)	4.89	.001
LDH (U/L)	385.7 ± 108.6	321.2 ± 85.4	3.85	.001

CRP = C-reactive protein, LDH = lactate dehydrogenase, MPP = Mycoplasma pneumoniae pneumonia, SMPP = severe Mycoplasma pneumoniae pneumonia, WBC = white blood cell count. Data are presented as mean ± standard deviation, n (%), or median (interquartile range).

### 3.2. Independent predictors of SMPP

Univariate logistic regression analysis showed that LDH (odds ratio [OR] = 1.43, 95% confidence interval [CI]: 1.18–1.86, *P* = .004), CRP (OR = 2.15, 95% CI: 1.82–2.93, *P* = .004), and serum NETs (OR = 3.40, 95% CI: 1.56–6.23, *P* = .001) were associated with the occurrence of SMPP (Table 2). After adjusting for potential confounding factors, multivariate logistic regression analysis confirmed that serum NETs (OR = 4.32, 95% CI: 3.45–7.25, *P* = .023), CRP (OR = 3.62, 95% CI: 1.85–9.25, *P* = .010), and LDH (OR = 1.63, 95% CI: 1.18–2.26, *P* = .001) were independent predictors of SMPP in children with MPP (Table 2).

**Table 2:** Univariate and multivariate logistic regression analyses for independent predictors of SMPP.

Variable	Univariate regression OR (95% CI)	*P*	Multivariate regression OR (95% CI)	*P*
WBC	1.12 (0.86–1.24)	.352	1.26 (0.42–2.34)	.825
LDH	1.43 (1.18–1.86)	.004	1.63 (1.18–2.26)	.001
CRP	2.15 (1.82–2.93)	.004	3.62 (1.85–9.25)	.010
NETs	3.40 (1.56–6.23)	.001	4.32 (3.45–7.25)	.023

CI = confidence interval, CRP = C-reactive protein, LDH = lactate dehydrogenase, NETs = neutrophil extracellular traps, OR = odds ratio, WBC = white blood cell count.

### 3.3. Correlation between serum NETs and CRP/LDH levels

**Figure 2.**
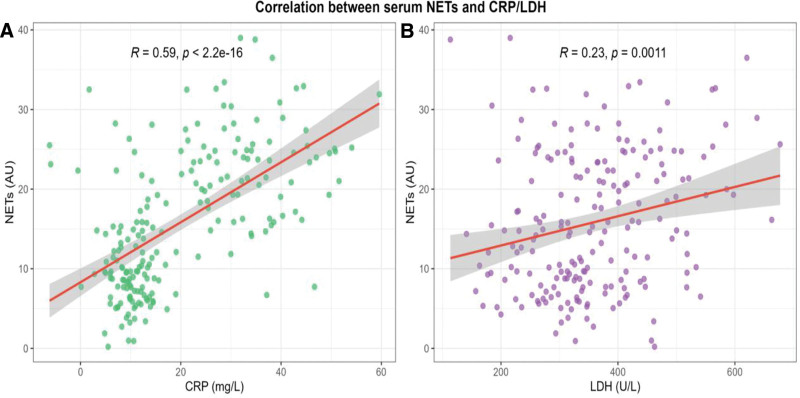
Correlation between NETs and CRP/LDH. CRP = C-reactive protein, LDH = lactate dehydrogenase, NETs = neutrophil extracellular traps.

Pearson correlation analysis was performed to explore the relationship between serum NETs and CRP, LDH levels. As shown in Figure 2, serum NETs were positively correlated with CRP (*r* = 0.59, *P* < 2.2e-16) and LDH (*r* = 0.23, *P* = .0011), indicating that the elevation of NETs was associated with the increase of traditional inflammatory markers and tissue damage indicators in children with MPP.

### 3.4. Predictive value of serum NETs, CRP, and LDH for SMPP

**Figure 3.**
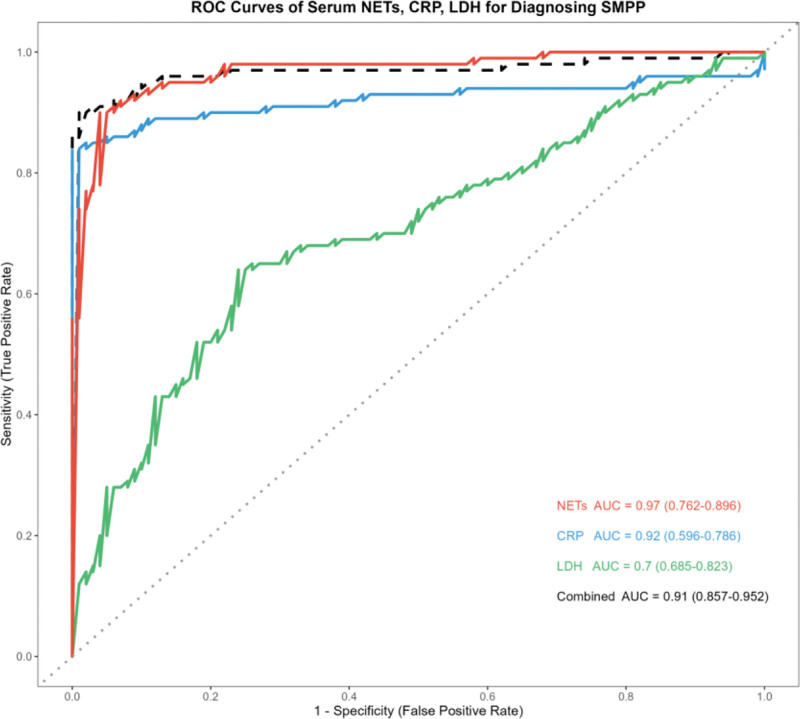
ROC curves of serum NETs, CRP, LDH for prediction of SMPP. CRP = C-reactive protein, LDH = lactate dehydrogenase, NETs = neutrophil extracellular traps, ROC = receiver operating characteristic curve, SMPP = severe Mycoplasma pneumoniae pneumonia.

Receiver operating characteristic (ROC) curve analysis was used to evaluate the predictive value of serum NETs, CRP, and LDH for SMPP. As shown in Table 3 and Figure 3, serum NETs had the highest area under the curve (AUC) among the 3 indicators (AUC = 0.83, 95% confidence interval [CI]: 0.762–0.896, *P* < .001), with a cutoff value of 19.2, corresponding sensitivity of 0.818 and specificity of 0.835. The AUC of LDH was 0.76 (95% CI: 0.685–0.823, *P* < .001), with a cutoff value of 312.5 U/L, sensitivity of 0.688 and specificity of 0.726. The AUC of CRP was 0.70 (95% CI: 0.596–0.786, *P* < .001), with a cutoff value of 10.2 mg/L, sensitivity of 0.628 and specificity of 0.753. Additionally, the combined detection of serum NETs, CRP, and LDH showed an AUC of 0.91 (95% CI: 0.857–0.952, *P* < .001), which was higher than the AUC of any single indicator.

**Table 3:** ROC curve of serum NETs, CRP, and LDH.

Factors	AUC	95% CI	Sensitivity	Specificity	Cut-off	*P*
Serum Nets	0.83	0.762–0.896	0.818	0.835	19.2	.001
CRP	0.70	0.596–0.786	0.628	0.753	10.2	.001
LDH	0.76	0.685–0.823	0.688	0.726	312.5	.001

Receiver operating characteristic (ROC) curve analysis of serum neutrophil extracellular traps (NETs), C-reactive protein (CRP) and lactate dehydrogenase (LDH) for predicting severe Mycoplasma pneumoniae pneumonia (SMPP) in children. AUC = area under the curve, CI = confidence interval.

## 4. Discussion

Mycoplasma pneumoniae pneumonia (MPP) is a common community-acquired respiratory infection in children, characterized by a 2 to 4 week incubation period and year-round prevalence, with endemic outbreaks occurring every 2 to 3 years in most regions of China.^[9]^ While MPP typically presents with nonspecific upper and lower respiratory tract symptoms and is self-limiting in most cases, a significant proportion of patients progress to severe mycoplasma pneumoniae pneumonia (SMPP) or refractory pneumonia. SMPP is often complicated by pleural effusion, multiple organ dysfunction syndrome, and long-term sequelae such as obstructive bronchiolitis and bronchiectasis; severe cases may develop respiratory failure and hypoxemia, requiring mechanical ventilation, extracorporeal membrane oxygenation, or other life-support interventions, and even leading to death.^[10,11]^ Against the backdrop of post-COVID-19 pandemic resurgence of MPP and increasing macrolide resistance,^[3,4,12]^ the early identification of SMPP and timely initiation of targeted treatment have become critical to improving patient prognosis, making the exploration of reliable severity-related biomarkers clinically imperative.Our findings confirm that serum NETs are significantly elevated in children with SMPP and exhibit superior predictive value compared with traditional markers CRP and LDH. These results build upon and extend our preliminary exploratory observations,^[13]^ which first suggested the potential of serum NETs for SMPP prediction. The present study provides definitive, methodologically complete evidence with a larger sample (n = 200), 1:1 group matching, multivariate logistic regression confirming independent predictive effects, and head-to-head ROC comparison of 3 biomarkers.

Neutrophil extracellular traps (NETs), web-like structures composed of decondensed chromatin and granular proteins released by activated neutrophils, play a dual role in infectious diseases: appropriate NET formation facilitates pathogen trapping and clearance, while excessive or dysregulated NET release (NETosis) triggers oxidative stress and inflammatory responses, leading to tissue and organ damage. Accumulating evidence has linked NETs to the pathogenesis of various respiratory infections, including bacterial and viral pneumonias.^[12,14]^ For instance, in COVID-19 patients with acute respiratory distress syndrome (ARDS), interleukin-1β (IL-1β) and NETs form a feedforward loop that directly damages endothelial cells and impairs endothelial barrier integrity^[15]^; Streptococcus pneumoniae evades neutrophil phagocytosis by inducing NET formation,^[16]^ and Pseudomonas aeruginosa-associated NETs in chronic lower respiratory tract infections are associated with increased sputum viscosity and impaired lung function^.[17]^ Our study extends these findings to MPP, demonstrating that serum NETs levels are significantly higher in children with SMPP than in those with non-severe MPP, and confirming NETs as an independent predictor of SMPP. Notably, the elevation of NETs in SMPP exhibits distinct characteristics compared to other pneumonias,suggesting that Mycoplasma pneumoniae may induce a more subtle yet pathogenic NETosis.

The pathological progression of MPP to SMPP is closely associated with an exaggerated inflammatory response and subsequent pulmonary and extrapulmonary complications. Our results show that serum C-reactive protein (CRP) and lactate dehydrogenase (LDH) levels are significantly elevated in the SMPP group, consistent with the “Expert Consensus on the Diagnosis and Treatment of Mycoplasma Pneumonia in Children (2023 Edition)” which highlights the value of these indicators in assessing MPP severity.^[1,3,18]^ Logistic regression analysis further confirmed CRP and LDH as independent predictors of SMPP. Moreover, Pearson correlation analysis revealed positive correlations between serum NETs and CRP as well as LDH, indicating that NETs elevation is closely associated with the increase in traditional inflammatory markers and tissue damage indicators, and may synergistically promote the progression of MPP to severity.

The localized lung injury in SMPP remains poorly understood, but our findings provide new insights into its underlying mechanisms. The higher NETs concentration in BALF from affected lungs compared to unaffected lungs suggests that excessive NET activation may contribute to localized pulmonary damage in SMPP. This is supported by previous studies: Kang et al reported a severe imbalance in the ratio of type 1 to type 2 helper T cells (Th1/Th2) in BALF of SMPP children,^[19]^ and Zheng et al demonstrated that NETs promote Th2-mediated signal transducer and activator of transcription 6 (STAT6)-dependent overexpression of complement C3.^[20]^ Collectively, these findings suggest that the NETs-mediated Th2-STAT6-C3-NETs cascade may be a key pathway driving localized lung injury in SMPP. As the first inflammatory mediators to reach sites of injury, NETs regulate both innate and adaptive immune responses, exert strong bactericidal effects, and promote inflammation^[21]^; they can also activate plasmacytoid dendritic cells to produce high levels of interferon-α via DNA-Toll-like receptor 9 (TLR9) signaling,^[22]^ and induce pyroptosis of alveolar macrophages by regulating nucleotide-binding oligomerization domain-like receptor protein 3 (NLRP3) deubiquitination, exacerbating sepsis-related lung injury.^[23]^ These mechanisms may further explain the role of NETs in SMPP pathogenesis.

ROC curve analysis underscores the clinical value of NETs, CRP, and LDH in predicting SMPP. Serum NETs exhibited the highest area under the curve among the 3 indicators, with superior sensitivity and specificity compared to LDH and CRP. Notably, the combined detection of NETs, CRP, and LDH yielded an even higher AUC of 0.91 indicating that the combination of these indicators can further improve the accuracy of SMPP prediction, providing a reliable laboratory basis for early clinical identification and severity assessment of pediatric SMPP.

This study has several limitations that should be acknowledged. First, as a single-center study conducted in East China, regional variations in Mycoplasma pneumoniae strains and host immune responses could affect the broader applicability of the results. Second, the study did not analyze specific NET components, which may limit our understanding of the precise mechanisms by which NETs contribute to SMPP. Despite these limitations, the study’s core findings remain valid and provide a foundation for future research.

## 5. Conclusion

In conclusion, overactive NETs in children with MPP may be associated with the development of SMPP and localized lung injury. Serum NETs, CRP, and LDH are independent predictive factors for SMPP, with NETs exhibiting superior predictive value compared to CRP and LDH. The combined detection of these indicators can enhance the accuracy of early SMPP identification, aiding in the formulation of targeted treatment plans and improving patient outcomes. Future studies should explore the specific mechanisms of NETs in SMPP pathogenesis and validate the findings in multi-center cohorts to further confirm the clinical utility of these biomarkers.

## Author contributions

**Conceptualization:** Baoxi Zhao, Junsheng Jiang.

**Data curation:** Baoxi Zhao, Junsheng Jiang.

**Formal analysis:** Baoxi Zhao, Junsheng Jiang.

**Funding acquisition:** Zhonghua Hu.

**Investigation:** Lina Qi, Junsheng Jiang.

**Methodology:** Lina Qi.

**Project administration:** Lina Qi, Lifen Yuan.

**Resources:** Lifen Yuan.

**Software:** Lifen Yuan.

**Supervision:** Lifen Yuan, Junsheng Jiang.

**Validation:** Lina Qi, Lifen Yuan, Baoxi Zhao, Junsheng Jiang.

**Visualization:** Lina Qi, Baoxi Zhao.

**Writing – original draft:** Lina Qi, Lifen Yuan.

**Writing – review & editing:** Zhonghua Hu, Junsheng Jiang.
